# The Anatomy of the Thoracic Duct and Cisterna Chyli: A Meta-Analysis with Surgical Implications

**DOI:** 10.3390/jcm13154285

**Published:** 2024-07-23

**Authors:** Dawid Plutecki, Michał Bonczar, Jakub Wilk, Sandra Necka, Miłosz Joniec, Ahmed Elsaftawy, Aleksandra Matuszyk, Jerzy Walocha, Mateusz Koziej, Patryk Ostrowski

**Affiliations:** 1 Collegium Medicum, Jan Kochanowski University, 25-369 Kielce, Poland; 2 Department of Anatomy, Jagiellonian University Medical College, 33-332 Kraków, Poland; 3 Youthoria—Youth Research Organization, 30-363 Kraków, Poland; 4 Chiroplastica—Lower Silesian Centre of Hand and Aesthetic Surgery, 54-117 Wrocław, Poland

**Keywords:** thoracic duct, cisterna chyli, anatomy, thorax, head and neck, surgery, lymphatic system

## Abstract

**Background**: The thoracic duct (TD) and the cisterna chyli (CC) exhibit a high degree of variability in their topographical and morphometric properties. **Materials and Methods**: PubMed, Scopus, Embase, Web of Science, Cochrane Library, and Google Scholar were searched to identify all studies that included information regarding the morphometric and topographical characteristics of the TD and CC. **Results**: The most frequent location of the TD termination was the left venous angle, with a pooled prevalence of 45.29% (95% CI: 25.51–65.81%). Moreover, the TD terminated most commonly as a single vessel (pooled prevalence = 78.41%; 95% CI: 70.91–85.09%). However, it divides into two or more terminating branches in approximately a quarter of the cases. The pooled prevalence of the CC was found to be 55.49% (95% CI: 26.79–82.53%). **Conclusions**: Our meta-analysis reveals significant variability in the anatomy of the TD and CC, particularly regarding TD termination patterns. Despite the predominance of single-vessel terminations, almost a quarter of cases exhibit branching, highlighting the complexity of the anatomy of the TD. These findings demonstrate the importance of detailed anatomical knowledge for surgeons to minimize the risk of accidental injury during head and neck, as well as thoracic surgeries. Our study provides essential insights that can enhance surgical safety and efficacy, ultimately improving patient outcomes.

## 1. Introduction

**Figure 1:**
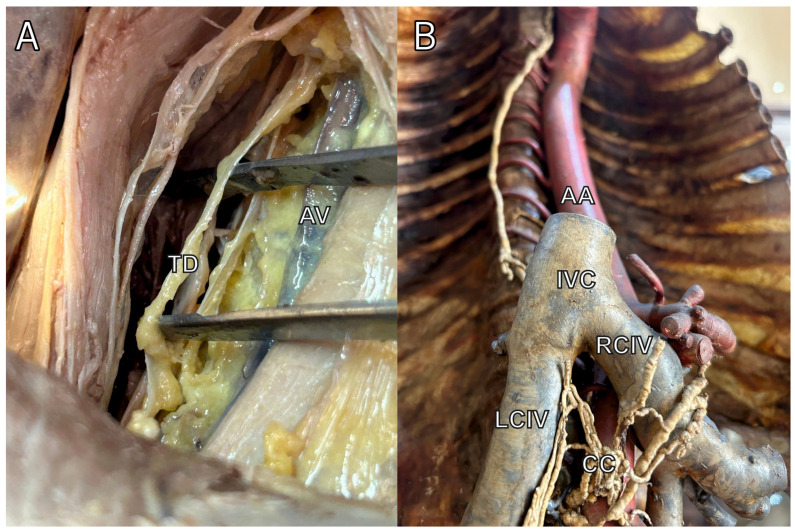
Photos of thoracic duct (TD) (**A**) and cisterna chyli (CC) (**B**) from the cadaveric dissection. AV—Azygos Vein. AA—Abdominal Aorta. IVC—Inferior Vena Cava. RCIV—Right Common Iliac Vein. LCIV—Left Common Iliac Vein.

The thoracic duct (TD) and the cisterna chyli (CC) are crucial components of the human lymphatic system (Figure 1). The TD, the largest lymphatic vessel in the body, originates from the convergence of several lymphatic trunks in the abdominal region. It often begins in the CC, a dilated sac located at the level of the second lumbar vertebra, although this structure is not present in all individuals [1,2]. From its origin, the TD ascends through the thorax, eventually emptying into the left venous angle, where the left internal jugular and left subclavian veins converge. This pathway allows the TD to drain lymph from the lower body, left thorax, left upper limb, and left side of the head and neck, enabling the return of lymphatic fluid to the bloodstream. The right upper quadrant of the body drains to the right venous angle, usually via a right lymphatic duct [3].

The TD and CC demonstrate a high level of variability that can significantly impact both clinical practices and surgical outcomes [4]. Variations in the origin, course, and termination of the TD are well documented. For instance, some individuals may have an absent CC, with direct lumbar trunk drainage into the TD [5]. However, most variations are seen in the area of the head and neck, where the TD terminates. Numerous termination patterns of the TD have been presented, such as the duct draining into the left subclavian vein or even the left brachiocephalic vein [6]. Furthermore, the TD may terminate into a variable number of branches, draining into one or more cervical veins.

Understanding the anatomical characteristics and variations of the TD is crucial because accidental injury to the TD can lead to complications that significantly decrease the patient’s quality of life. During radical neck dissections, TD injury and subsequent chylous leakage may occur in 1.0% to 3.0% of patients [6]. During esophageal resections, reconstructions, or thoracic surgeries, TD damage may lead to chylothorax, with a prevalence of up to 8.0% [7]. Chylous fistula or chylothorax can cause serious systemic complications, including malnutrition, immunosuppression, and respiratory distress [7]. Therefore, surgeons performing procedures that risk TD injury must have a thorough understanding of TD anatomy to minimize accidental damage.

Several reviews have addressed the anatomical characteristics of the TD, but no comprehensive meta-analysis has been published to demonstrate the major anatomical features of the TD and CC based on all available data. The primary objective of this meta-analysis was to provide surgeons with crucial data on the topographical and morphometric properties of the TD and CC. We hope that the results of our study will help reduce the risk of potential injury to the TD during various head and neck, as well as thoracic, surgeries.

## 2. Methods

### 2.1. Search Strategy

Medical databases such as PubMed, Embase, Scopus, and Web of Science were searched to gather all studies on anatomical characteristics, morphometry, variations, and relationship to the surrounding structures of the TD and CC. The data collection for this study ended in January 2024. In agreement with the Boolean technique, the following search terms were employed: ((thoracic duct) OR (Hoorne’s canal)) AND (anatomy). The search terms were individually adapted to each database to minimize potential bias. Neither date, language, article type, nor text availability conditions were applied. An additional search was conducted through the references of the identified studies at the end of the search stage to ensure the accuracy of the process. During this study, the Preferred Reporting Items for Systematic Reviews and Meta-analyses (PRISMA) guidelines were followed. Furthermore, the Critical Appraisal Tool for Anatomical Meta-analysis (CATAM) was used to provide the highest-quality findings [8].

### 2.2. Eligibility Assessment

The database search and the manual search identified a total of 10,940 studies that were initially evaluated by two independent reviewers. After removing duplicates and irrelevant records, a total of 191 articles were qualified for a full-text evaluation. To minimize potential bias and maintain accurate statistical methodology, articles such as case reports, case series, conference reports, reviews, letters to editors, and studies that provided incomplete or irrelevant data were excluded. The inclusion criteria consisted of original studies with extractable numerical data regarding the topic of this study. Studies written in languages other than English were also evaluated but only included in case of high certainty of maintaining the meaning of the extracted data. Finally, a total of 41 studies were included in this meta-analysis. Additionally, the AQUA Tool, which was specifically designed for anatomical meta-analyses, was used to minimize the potential bias of included studies [9,10].

### 2.3. Data Extraction

Data from qualified studies were extracted by two independent reviewers. Qualitative data, such as year of publication, country and continent of origin, data collection methodology, and information on diseases in the studied groups, were collected. Quantitative data, such as sample size, numerical data on anatomical characteristics, morphometry, and relationship with the anatomical surroundings of the TD and CC, were also gathered. Any discrepancies between studies identified by the two reviewers were resolved by contacting the authors of the original studies whenever possible or by consensus with a third reviewer.

### 2.4. Statistical Analysis

To perform the meta-analyses, STATISTICA version 13.1 software (StatSoft Inc., Tulsa, OK, USA), MetaXL version 5.3 software (EpiGear International Pty Ltd., Wilston, QLD, Australia), and Comprehensive Meta-analysis version 4.0 software (Biostat Inc., Englewood, NJ, USA) were used. A random-effects model was used in all analyses. The heterogeneity among the studies was evaluated, using both the Chi-squared test and the I-squared statistic [11]. The I-squared statistic was interpreted as follows: 0–40% as “might not be important”; 30–60% as “may represent moderate heterogeneity; 50–90% as “may represent substantial heterogeneity”; and 75–100% as “may represent considerable heterogeneity”. *p*-value of <0.05 and the confidence intervals (95% CI) were used to find statistically significant differences between the studied groups. In the case of overlapping confidence intervals, differences were considered statistically insignificant.

## 3. Results

### 3.1. Search Results

**Figure 2:**
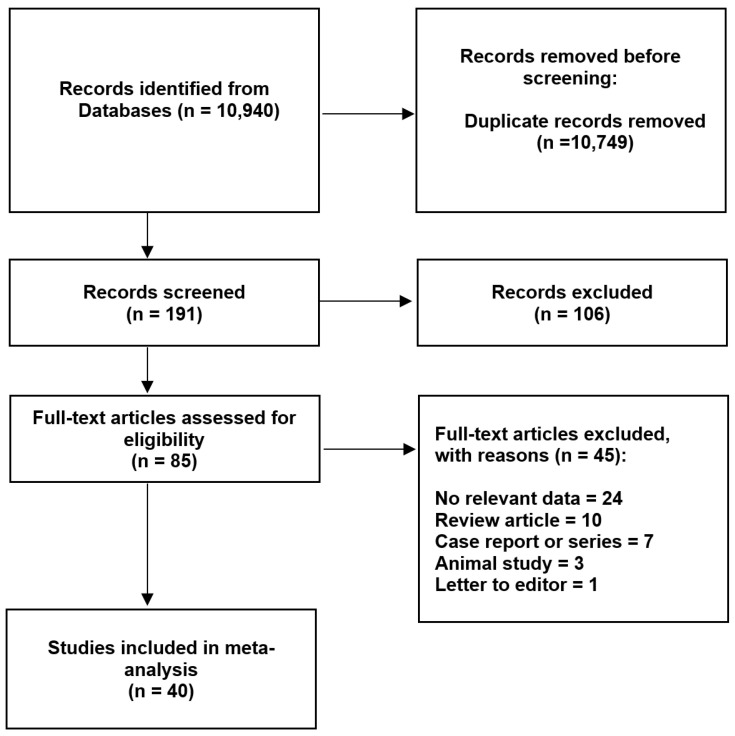
Flow diagram presenting process of collecting data included in this meta-analysis.

After the selection of the initially accepted 85 studies, a total of 45 studies were excluded. Most of them (n = 24) were disqualified due to the lack of relevant data. Eventually, a total of 40 studies were included in this meta-analysis [2,12,13,14,15,16,17,18,19,20,21,22,23,24,25,26,27,28,29,30,31,32,33,34,35,36,37,38,39,40,41,42,43,44,45,46,47,48,49,50]. An overall data collection process is presented in Figure 2. The characteristics of all the submitted studies are presented in Table 1.

### 3.2. Thoracic Duct Location of Termination

A total of 1334 TDs were assessed in this part of the analysis. The most common location of termination of the TDs was found to be the left internal jugular–subclavian joint with the pooled prevalence established at 45.29% (95% CI: 25.51–65.81%). The pooled prevalence of TDs terminating at the left internal jugular vein was set to be 24.19% (95% CI: 13.18–37.16%). The pooled prevalence of TDs terminating at the left subclavian vein was found to be 16.32% (95% CI: 8.40–26.12%). Detailed results regarding this part of the analysis can be found in Table 2.

### 3.3. Thoracic Duct Termination Type

**Figure 3:**
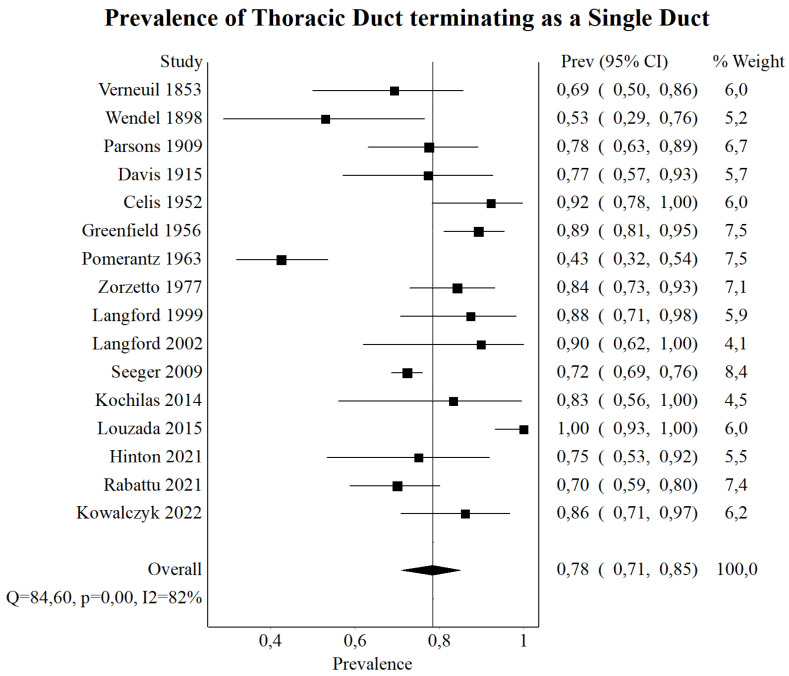
Forrest plot—analysis of the prevalence of thoracic duct terminating as a single duct [13,14,15,19,21,22,23,28,31,33,34,38,40,42,46,47].

A total of 1091 TD were assessed in this part of the analysis. The pooled prevalence of TD terminating as a single duct was found to be 78.41% (95% CI: 70.91–85.09%). The pooled prevalence of TD terminating as a bifid or double duct was set to be 13.76% (95% CI: 10.10–17.87%). Detailed results regarding this part of the analysis can be found in Table 3 and Figure 3.

### 3.4. Cisterna Chyli

**Figure 4:**
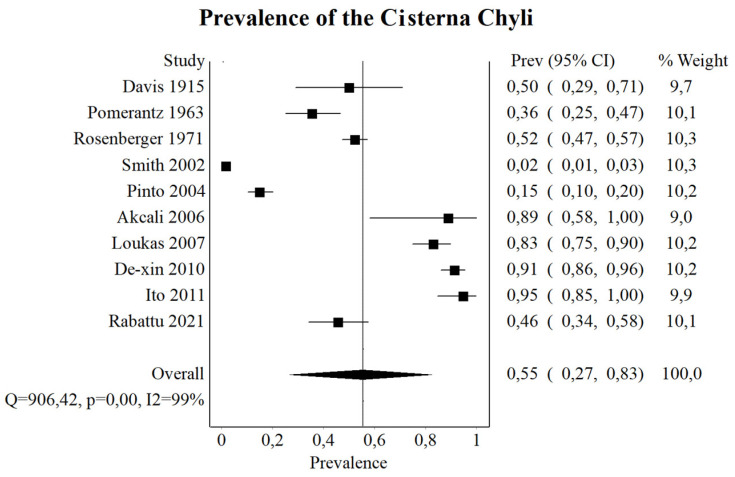
Forrest plot—analysis of the prevalence of cisterna chyli [2,29,32,33,34,35,39,45,47,49].

The pooled prevalence of the CC, based on the 1447 specimens, was set to be 55.49% (95% CI: 26.79–82.53%). Furthermore, the most common location of the CC was the L1–L2 vertebral levels with a pooled prevalence established at 19.08% (95% CI: 2.60–43.33%). The pooled mean length of the CC was set to be 18.25 mm (SE = 1.89). Detailed results regarding this part of the analysis can be found in Table 4 and Figure 4.

### 3.5. Thoracic Duct Diameter

A total of 471 TDs were assessed in this part of the analysis. The pooled mean TD diameter, based on the results of the computed tomography analyses, was found to be 3.79 mm (SE = 0.78). Detailed results regarding this part of the analysis can be found in Table 5.

## 4. Discussion

The anatomy of the TD and CC demonstrates considerable variability. While there have been previous reviews on the TD, unfortunately, the description of the anatomy of the TD and CC is often obscured in the available literature and is not reported as a set of specific types. Moreover, studies that have attempted to categorize the variations in the anatomy of the TD and CC have been inconsistent. Therefore, a systematic search of the literature and a meta-analysis were performed to clarify and conclude the variations in the anatomy and dimensions of these structures.

The human lymphatic system begins its development at the end of the sixth gestational week [51]. During the seventh and eighth gestational weeks, the TD forms from two large vessels located anterior to the aorta, which connect the paired jugular lymph sacs superiorly to the CC. These vessels subsequently enlarge and fuse to form the embryonic right and left TDs, with multiple communications between them [39]. The lower two-thirds of the adult TD originates from the right embryonic duct, while the upper third is derived from the left embryonic TD [47,52]. Variations in the course of the TD are common and can result from the persistence or failure of this developmental pattern to progress in the typical manner [5].

The cervical segment of the TD may be the most variable portion of this major lymphatic vessel. It is situated at the base of the neck on the left side. It is bordered anteriorly by the carotid sheath, medially by the esophagus, laterally by the omohyoid muscle, and posteriorly by the prevertebral fascia. In this region, the TD typically extends 3 to 5 cm above the clavicle before descending and moving anteriorly over the subclavian artery. It terminates within 1 cm of the left venous angle [53]. In this area, the TD exhibits variations in both the location of its termination and the number of terminating branches. Numerous termination patterns have been described in the literature and major anatomical textbooks, including the left venous angle, the left internal jugular vein, the left subclavian vein, and even the left external jugular vein, amongst others [3,14,27,33]. The overall frequencies of these terminations vary substantially between the studies and the reviews present in the literature. A review by Phang et al. [5] found that the TD most commonly terminates into the internal jugular vein (46.00%), with more than a quarter of cases terminating at the left venous angle (32.00%). Similarly, Bellier et al. [6] reported the internal jugular vein as the most frequent termination site (54.00%). However, this finding primarily relies on data from Seeger et al. [31], where the exact termination location of the TD was not specified and only the number of terminating branches was noted. Therefore, the results of the review conducted by Bellier et al. [6] regarding the location of the TD termination may be inaccurate.

**Figure 5:**
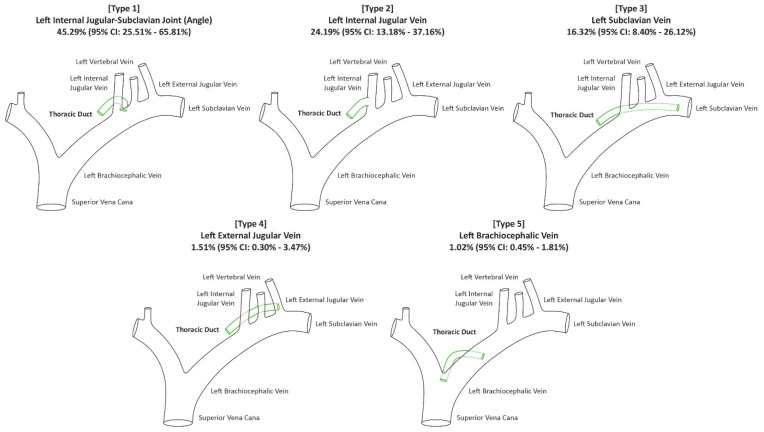
Illustration of the main types of location of termination of the thoracic duct.

Due to inconsistencies in the reported frequencies of TD termination locations, we developed a novel classification system consisting of six types, as illustrated in Figure 5. Type 1 represents the TD terminating at the left venous angle, which occurs in 45.29% of cases. Type 2 showcases a termination at the left internal jugular vein, observed in 24.19% of cases. Type 3 demonstrates the TD ending in the subclavian vein, with a prevalence of 16.32%. The last three types are rarer: Type 4 involves termination at the left external jugular vein (1.51%) and Type 5 involves termination at the left brachiocephalic vein (1.02%). Type 6 encompasses the rarest termination types, with a prevalence of less than one percent, including the TD ending in the left vertebral vein and the right jugular venous angle, among others. This classification system highlights the significant variability in TD termination locations. However, it is important to note that the TD predominantly terminates in the region of the left internal jugular and left subclavian veins, with Types 1, 2, and 3 accounting for 85.80% of cases.

Prior to its termination, the TD may branch into several terminating vessels of varying numbers. Most studies indicate that the TD typically terminates as a single vessel without branching [31,46,47]. However, in a cadaveric study conducted by Shimada and Sato [30], it was reported that the TD terminated into multiple branches rather than reaching the terminating vein as a single vessel. Our meta-analysis demonstrates that the TD terminates as a single vessel in the majority of cases (78.41%). However, in a notable number of cases, the TD divides into two vessels before termination (13.76%) and, rarely, into three or more vessels (8.54%). The complexity of the termination pattern of the TD in the cervical region showcases the need for head and neck surgeons to exercise caution when operating in this area. The potential for the TD to divide into two or more branches in almost a quarter of cases increases the risk of injury to these critical lymphatic structures.

The CC is said to be the origin of the TD, and it receives lymph from the intestinal lymphatic trunk, the left lumbar trunk, and, more rarely, the right lumbar trunk [5]. It receives smaller contributions from the periaortic nodes and intercostal lymphatics [39]. However, its overall prevalence is controversial, with some studies reporting that the CC is present in the majority of the subjects [2,39], while others state that it is prevalent in less than half [29]. When the CC is absent, the TD is formed by the merging of a less distinct plexus of lymphatic channels [5]. To clear the confusion regarding the overall frequency of the CC, we analyzed all of the numerical data concerning this topic in the available literature. The results of the present meta-analysis showcase that the pooled prevalence of the CC is 55.49%. Moreover, the topography and morphometric properties of the CC were analyzed. Interestingly, the CC was located most commonly in the transitioning area between the thoracic and lumbar vertebrae (T12–L2), and the mean length of the CC was found to be 18.25 mm. This demonstrates that the origin of the TD is significantly variable, as the CC is only present in half of the individuals. Surgeons need to be aware of the prevalence and topography of the CC as iatrogenic damage to this lymphatic structure during abdominal dissection may lead to chylous ascites or chylothorax [54].

Chyle leak is a rare but serious complication following various surgical procedures in both the thoracic and cervical regions, with prevalences ranging between 1.0% and 9.0% [7,53,54]. The subsequent systemic complications that may occur due to chyle leak include hypovolemia due to the high pressure of outflow, electrolyte abnormalities, and immunosuppression due to the loss of immunoglobulins and T lymphocytes that are present in the lymph [7]. If left untreated, the mortality rates may be as high as 50% [55]. Due to the close anatomical relationship of the TD to the esophagus, the lymphatic vessel is at significant risk of injury during esophagectomies [56]. The high rate of variations in the anatomy of the TD has been thought to be linked with an increased risk of chyle leaks, as well as low body mass index and neoadjuvant chemoradiotherapy [48,54,57,58]. In order to reduce the risk of this serious complication, the surgeon needs to possess adequate knowledge regarding the topography of the TD.

The distal part of the TD, near its termination, is also at risk during radical neck dissection, including level IV, as well as during thyroidectomies and lateral neck dissection [53,59]. Similarly to esophagectomies, anatomical variations of the TD increase the risk of accidental injury of this structure during the aforementioned procedures [53]. Knowledge of the complete anatomy of the TD is of utmost importance to both reduce the risk of accidental damage to the vessel and also to aid in the surgical treatment of the complication that already occurred. The operative treatments for chyle leak include, amongst others, video-assisted thoracoscopic duct ligation and percutaneous lymphangiography-guided thoracic duct cannulation and embolization [60]. Here, the physician is not only required to have knowledge about the topography of the vessel but also the normal morphometric properties of the TD and the CC, especially for interventionists. The results of the present study demonstrate that the mean diameter at the termination of the TD is 3.79 mm (based on CT scans), and the morphometric and the mean length of the CC was 18.25 mm. These data may be of significance during the TD embolization, mainly in the intravascular navigation through the central lymphatic network. Previous studies have stated that the most technically challenging step of this procedure is being able to access the central lymphatic system, namely the CC or the lumbar lymphatic trunks [60].

This study is not without limitations. It may be burdened with potential bias, as the accuracy of the data taken from various publications limits the results of this meta-analysis. A potential sexual dimorphism in the anatomical characteristics of TD and CC was not established due to the lack or inconsistency of data. Analogically, no gender-related statistics were enrolled. Moreover, an analysis of morphometrical features of the TD and CC was not enrolled in relation to the height of the subjects due to the lack of such information in primary studies. Furthermore, the data regarding the lymphatics of the right upper quadrant and the location of the TD branches before their termination are also limited in the literature. Those deficits should be considered in further studies. Furthermore, a number of tributaries and variations along the intrathoracic course of the TD also were not analyzed due to an insufficient amount of data in the literature. Even though only the patients without visible and relevant comorbidities were analyzed, it must be noted that mutations in genes such as NRAS, KRAS, and PIK3CA may influence anatomical variations of the thoracic duct. Although not without limitations, our meta-analysis attempts to estimate TD and CC anatomy based on the data from the literature that meet the requirements of evidence-based medicine [61,62].

## 5. Conclusions

The anatomy of the TD is highly variable, particularly in its termination within the cervical region. Due to the inconsistencies in the reported locations of TD termination, we developed a novel classification system. Type 1 (termination at the left venous angle) was the most frequently observed, with a pooled prevalence of 45.29%. Additionally, the TD most commonly terminates as a single vessel (78.41%), though it divides into two or more branches in nearly a quarter of cases. The CC also exhibited significant variability, being present in approximately 55.49% of the analyzed cohort. This high rate of anatomical variation increases the risk of accidental damage to the TD during various head and neck surgeries, such as radical neck dissections (including level IV), thyroidectomies, and lateral neck dissections. Our findings are also relevant for surgeons performing esophagectomies. It is hoped that the results of this meta-analysis may aid in reducing the risk of inadvertent injury to the TD and CC, thereby improving the safety and efficiency of surgical procedures involving the head and neck, as well as the thorax.

## Figures and Tables

**Table 1:** Characteristics of included studies.

**Adachi 1928 [50], Japan, Asia**	
Methods	Cadaveric dissection
Participants	261 cadavers
Outcomes	Location of termination
**Akcali 2006 [49], Turkey, Asia**	
Methods	Cadaveric dissection
Participants	9 male cadavers
Outcomes	Location of termination, cisterna chyli location and prevalence and diameter and length
**Amore 2016 [48], Argentina, South America**	
Methods	Cadaveric dissection
Participants	12 cadavers (8 male and 4 female) Age range: 50–80 years
Outcomes	Location of termination
**Archimbaud 1969 [24], France, Europe**	
Methods	Cadaveric dissection
Participants	40 cadavers
Outcomes	Location of termination
**Celis 1952 [13], Mexico, North America**	
Methods	Cadaveric dissection
Participants	26 cadavers
Outcomes	Termination of TD, location of termination
**Correia 1925 [20], Portugal, Europe**	
Methods	Cadaveric dissection
Participants	22 cadavers
Outcomes	Location of termination
**Davis 1915 [47], USA, North America**	
Methods	Cadaveric dissection
Participants	22 cadavers
Outcomes	Termination of TD, location of termination, diameter of TD, cisterna chyli location, and prevalence
**De-xin 2010 [2], China, Asia**	
Methods	Radiological study Imaging: MRI
Participants	139 volunteers (96 males and 43 females) Age range: 16–83 years
Outcomes	Diameter of TD, cisterna chyli location and prevalence and diameter and length
**Greenfield 1956 [19], USA, North America**	
Methods	Cadaveric dissection
Participants	75 cadavers
Outcomes	Termination of TD, location of termination
**Hinton 2021 [46], New Zealand, Australia, and Oceania**	
Methods	Radiological study Imaging: USG
Participants	20 volunteers
Outcomes	Termination of TD, diameter of TD
**Ito 2011 [45], Japan, Asia**	
Methods	Radiological study Imaging: MRI
Participants	38 volunteers
Outcomes	Diameter of TD, cisterna chyli prevalence
**Jacobbson 1972 [18], Sweden, Europe**	
Methods	Cadaveric dissection
Participants	100 cadavers
Outcomes	Location of termination
**Jdanov 1959 [26], France, Europe**	
Methods	Cadaveric dissection
Participants	100 cadavers
Outcomes	Location of termination
**Kammerer 2016 [44], Germany, Europe**	
Methods	Radiological study Imaging: CT
Participants	197 volunteers (131 males and 66 females) Age range: 12–89 years
Outcomes	Diameter of TD
**Kiyonaga 2012 [43], Japan, Asia**	
Methods	Radiological study Imaging: CT
Participants	50 volunteers (20 females and 30 males) Age range: 32–81 years
Outcomes	Cisterna chyli location and diameter and length
**Kochilas 2014 [15], USA, North America**	
Methods	Radiological study Imaging: USG
Participants	12 volunteers
Outcomes	Termination of TD
**Kowalczyk 2022 [42], Poland, Europe**	
Methods	Radiological study Imaging: USG
Participants	31 volunteers (16 females and 15 males) Mean age: 24 years
Outcomes	Termination of TD, location of termination, diameter of TD
**Kurylcio 1972 [17], Poland, Europe**	
Methods	Cadaveric dissection
Participants	50 cadavers
Outcomes	Location of termination
**Langford 1999 [40], UK, Europe**	
Methods	Cadaveric dissection
Participants	10 cadavers
Outcomes	Termination of TD, location of termination, diameter of TD
**Langford 2002 [14], UK, Europe**	
Methods	Cadaveric dissection
Participants	24 cadavers
Outcomes	Termination of TD, location of termination
**Liu 2006 [41], USA, North America**	
Methods	Radiological study Imaging: CT
Participants	301 volunteers (131 males and 170 females) Age range: 11–92 years
Outcomes	Diameter of TD
**Loukas 2007 [39], USA, North America**	
Methods	Cadaveric dissection
Participants	100 cadavers (44 females and 76 males) Age range: 55–86 years
Outcomes	Cisterna chyli location and prevalence and diameter and length
**Louzada 2015 [38], Brazil, South America**	
Methods	Cadaveric dissection
Participants	25 cadavers Mean age: 71 ± 15.3 years
Outcomes	Termination of TD, location of termination
**Niggeman 2010 [37], Germany, Europe**	
Methods	Radiological study Imaging: MRI
Participants	10 volunteers (4 females and 6 males) Mean age: 59
Outcomes	Cisterna chyli location
**Okuda 2009 [36], Japan, Asia**	
Methods	Radiological study Imaging: MRI
Participants	78 volunteers (69 males and 9 females) Age range: 25–82 years
Outcomes	Location of termination
**Parsons 1909 [21], UK, Europe**	
Methods	Cadaveric dissection
Participants	40 cadavers
Outcomes	Termination of TD, location of termination
**Pinto 2004 [35], Portugal, Europe**	
Methods	Cadaveric dissection and radiological study Imaging: MRI
Participants	200 volunteers
Outcomes	Cisterna chyli prevalence
**Pomerantz 1963 [34], USA, North America**	
Methods	Radiological study Imaging: X-ray
Participants	90 volunteers
Outcomes	Termination of TD, cisterna chyli prevalence
**Rabattu 2021 [33], France, Europe**	
Methods	Cadaveric dissection
Participants	70 cadavers (35 males and 45 females) Mean age: 80–92 years
Outcomes	Termination of TD, location of termination, cisterna chyli prevalence
**Rocca-Rosset 1961 [25], Italy, Europe**	
Methods	Cadaveric dissection
Participants	16 cadavers
Outcomes	Location of termination
**Rosenberger 1971 [32], USA, North America**	
Methods	Radiological study Imaging: X-ray
Participants	390 volunteers Age range: 0–70, >70 years
Outcomes	Cisterna chyli location and prevalence
**Seeger 2009 [31], Germany, Europe**	
Methods	Radiological study Imaging: USG
Participants	564 volunteers Age range: 17–87 years
Outcomes	Termination of TD
**Shafiroff 1959 [27], USA, North America**	
Methods	Cadaveric dissection
Participants	30 cadavers
Outcomes	Location of termination
**Shimada 1997 [30], Japan, Asia**	
Methods	Cadaveric dissection
Participants	100 cadavers (57 males and 43 females)
Outcomes	Location of termination
**Smith 2002 [29], USA, North America**	
Methods	Radiological study Imaging: CT
Participants	403 volunteers
Outcomes	Cisterna chyli location and diameter and length and prevalence
**Verneuil 1853 [22], France, Europe**	
Methods	Cadaveric dissection
Participants	26 cadavers
Outcomes	Termination of TD
**Wendel 1898 [23], Germany, Europe**	
Methods	Cadaveric dissection
Participants	17 cadavers
Outcomes	Termination of TD
**Xie 1987 [16], China, Asia**	
Methods	Observational study
Participants	20 cadavers
Outcomes	Location of termination
**Yalakurthi 2013 [12], India, Asia**	
Methods	Cadaveric dissection
Participants	41 cadavers
Outcomes	Location of termination
**Zorzetto 1977 [28], Brazil, South America**	
Methods	Cadaveric dissection
Participants	51 cadavers
Outcomes	Termination of TD, location of termination

**Table 2:** Statistical results of this meta-analysis regarding the location of termination of the thoracic duct. * Others included Right Internal Jugular Vein, left vertebral vein, Left Suprascapular Vein, Cervical Lymphatic Chain, Left Jugulovertebral Angle, or a complex configuration. LCI—lower confidence interval. HCI—higher confidence interval. Q—Cochran’s Q.

Category	N	Pooled Prevalence	LCI	HCI	Q	I^2^
*Overall*						
Left Internal Jugular–Subclavian Joint (Angle) [Type 1]	1334	45.29%	25.51%	65.81%	1274.79	98.12
Left Internal Jugular Vein [Type 2]		24.19%	13.18%	37.16%	601.40	96.01
Left Subclavian Vein [Type 3]		16.32%	8.40%	26.12%	434.32	94.47
Left External Jugular Vein [Type 4]		1.51%	0.30%	3.47%	111.42	78.46
Left Brachiocephalic Vein [Type 5]		1.02%	0.45%	1.81%	32.06	25.14
Other * [Type 6]		2.54%	1.12%	4.46%	77.25	68.93
*Europe*						
Left Internal Jugular–Subclavian Joint (Angle) [Type 1]	507	49.20%	25.31%	73.27%	279.82	96.43
Left Internal Jugular Vein [Type 2]		31.80%	14.53%	51.87%	193.84	94.84
Left Subclavian Vein [Type 3]		11.65%	4.00%	22.19%	92.69	89.21
Left External Jugular Vein [Type 4]		0.56%	0.06%	1.44%	6.63	0.00
Left Brachiocephalic Vein [Type 5]		1.05%	0.12%	2.68%	16.53	39.50
Other * [Type 6]		1.36%	0.25%	3.18%	16.99	41.15
*Asia*						
Left Internal Jugular–Subclavian Joint (Angle) [Type 1]	504	64.37%	18.39%	99.14%	353.72	98.59
Left Internal Jugular Vein [Type 2]		9.79%	0.00%	28.50%	136.55	96.34
Left Subclavian Vein [Type 3]		12.07%	0.38%	32.49%	108.72	95.40
Left External Jugular Vein [Type 4]		2.81%	0.00%	11.94%	85.90	94.18
Other * [Type 6]		1.30%	0.00%	4.03%	18.07	72.32

**Table 3:** Statistical results of this meta-analysis regarding the termination of the thoracic duct. LCI—lower confidence interval. HCI—higher confidence interval. Q—Cochran’s Q.

Category	N	Pooled Prevalence	LCI	HCI	Q	I^2^
*Overall*						
Single Duct	1091	78.41%	70.91%	85.09%	84.60	82.27
Bifid/Double Duct		13.76%	10.10%	17.87%	33.52	55.26
Plexiform (3 or more ducts)		8.54%	4.84%	13.13%	59.77	74.90
*Europe*						
Single Duct	780	74.27%	68.54%	79.61%	10.72	34.70
Bifid/Double Duct		18.15%	15.53%	20.93%	3.70	0.00
Plexiform (3 or more ducts)		9.37%	5.89%	13.54%	11.20	37.51
*North America*						
Single Duct	225	78.41%	49.38%	98.01%	65.18	93.86
Bifid/Double Duct		12.22%	4.85%	22.08%	12.71	68.54
Plexiform (3 or more ducts)		8.68%	0.81%	21.86%	24.58	83.73

**Table 4:** Statistical results of this meta-analysis regarding the cisterna chyli. LCI—lower confidence interval. HCI—higher confidence interval. Q—Cochran’s Q.

**Category**		**N**	**Pooled Prevalence**	**LCI**	**HCI**	**Q**	**I^2^**
*Prevalence of the Cisterna Chyli*							
Prevalence		1447	55.49%	26.79%	82.53%	906.42	99.01
*Location of the Cisterna Chyli*							
T9–T10		506	0.60%	0.03%	1.68%	7.88	11.19
T10–T11			0.89%	0.00%	2.89%	12.81	45.34
T11			2.35%	0.00%	7.02%	28.22	75.19
T11–T12			1.81%	0.00%	6.01%	26.79	73.87
T12			10.13%	0.18%	28.33%	136.88	94.89
T12–L1			15.60%	3.11%	33.83%	115.32	93.93
L1			13.12%	0.00%	34.72%	213.24	96.72
L1–L2			19.08%	2.60%	43.33%	177.72	96.06
L2			4.77%	0.00%	13.12%	72.99	90.41
L2–L3			0.71%	0.00%	2.23%	10.17	31.18
**Category**	**Mean**	**Standard Error**	**Variance**	**Lower Limit**	**Upper Limit**	**Z-Value**	***p*-Value**
*Length of the Cisterna Chyli*							
Length [mm]	18.25	1.89	3.56	14.55	21.94	9.68	0.00

**Table 5:** Statistical results of this meta-analysis regarding the diameter of the thoracic duct. CT—computed tomography. MRI—Magnetic Resonance Imaging. US—Ultrasound. (*) No new results were pooled in those categories due to the insufficient amount of data found in the literature; however, they are provided in this table due to potential clinical significance. All of the results are presented in millimeters [mm].

**Category**	**Mean**	**Standard Error**	**Variance**	**Lower Limit**	**Upper Limit**	**Z-Value**	***p*** **-Value**
Thoracic Duct Diameter at its Termination [CT]	3.79	0.78	0.61	2.26	5.33	4.85	0.00
**Category**	**Mean**			**Standard Error**			
Thoracic Duct Diameter at its Termination [US] (Hinton et al., 2021 [46]) *	1.7			0.575			
Thoracic Duct Diameter at its Termination [MRI] (Ito et al., 2011 [45]) *	3.37			1.25			
Thoracic Duct Diameter at its Termination [Cadavers] (Langford et al., 1999 [40]) *	5.3			1.625			

## Data Availability

The data that support the findings of this study are available from the corresponding author upon reasonable request.
